# The relationship between nomophobia and the distraction associated with smartphone use among nursing students in their clinical practicum

**DOI:** 10.1371/journal.pone.0202953

**Published:** 2018-08-27

**Authors:** Gabriel Aguilera-Manrique, Verónica V. Márquez-Hernández, Tania Alcaraz-Córdoba, Genoveva Granados-Gámez, Vanesa Gutiérrez-Puertas, Lorena Gutiérrez-Puertas

**Affiliations:** 1 Department of Nursing, Physiotherapy and Medicine, Faculty of Health Sciences, University of Almería, Almería, Spain; 2 Research Group for Health Sciences CTS-451, University of Almería, Almería, Spain; Nord University, NORWAY

## Abstract

**Background:**

The increasing concern about the adverse effects of overuse of smartphones during clinical practicum implies the need for policies restricting smartphone use while attending to patients. It is important to educate health personnel about the potential risks that can arise from the associated distraction.

**Objective:**

The aim of this study was to analyze the relationship between the level of nomophobia and the distraction associated with smartphone use among nursing students during their clinical practicum.

**Methods:**

A cross-sectional study was carried out on 304 nursing students. The nomophobia questionnaire (NMP-Q) and a questionnaire about smartphone use, the distraction associated with it, and opinions about phone restriction policies in hospitals were used.

**Results:**

A positive correlation between the use of smartphones and the total score of nomophobia was found. In the same way, there was a positive correlation between opinion about smartphone restriction polices with each of the dimensions of nomophobia and the total score of the questionnaire.

**Conclusions:**

Nursing students who show high levels of nomophobia also regularly use their smartphones during their clinical practicum, although they also believe that the implementation of policies restricting smartphone use while working is necessary.

## Introduction

The use of mobile phones has increased exponentially in the last decade [1]. In 2016, the number of mobile devices on a global level was estimated at 7.1 billion, more than the population of our planet [2]. These devices have become an essential part of our lives, due to their many functions, such as internet access, social networks, email, messaging, calling, playing games, and online shopping [3–5]. In the clinical environment, it has been demonstrated that smartphones may enhance the clinical practicum [6], experience and the nursing students’ learning [7].

Nevertheless, smartphones have been designated as potentially addictive and harmful to individuals’ professional and family life. They produce tolerance, which is to say, users have a growing need to increase the amount of time on their phones in order to feel satisfied, and they provoke habits of incessant, compulsive checking [8], which can have negative effects on daily life [9]. This addiction to mobile phones has given rise to the term nomophobia, defined as an uncontrollable fear of leaving home without a mobile phone. The term is an abbreviation of the expression, “no-mobile-phone-phobia" [10,11]. Nomophobia is an emerging human behavior phenomenon stemming from widespread mobile phone use [8,12], which causes symptoms such as: anxiety, emotional instability, aggressiveness and difficulty concentrating [13]. There are very few studies that address this problem, which is progressively growing [12]. One study carried out in the United Kingdom, showed that 66% of smartphone users suffer from nomophobia, with a higher rate among young people between 18–24 years old, followed by those between 25–34, and more prevalent among women than men [10]. Another study determined a prevalence of 42.6% among university students [14].

On the other hand, an increase in smartphone use among healthcare personnel has also been observed, for activities unrelated to the clinical environment [3,15–17]. Studies show that around 75% of nurses admit using a smartphone for personal communication while they work [15]. This increase in the use of smartphones could be related to behaviors consistent with nomophobia [18]. In addition, this overuse has consequences, such as putting off important tasks such as medical attention, and creates a source of great distraction [9] when being used for personal purposes [4,5].

The distraction of health personnel due to smartphone use, has been shown to lead to a lack of attention and a diminished capacity to remember important information [19]. This could cause adverse effects, such as threats to the safety of patients [20] loss of privacy and confidentiality of personal data and impaired communication between personnel and patients [21,22–24]. More specifically, smartphones have numerous resources, such as high-resolution cameras, that allow personnel to take photos, or record video or audio that can be shared instantly through networks or applications [25]. Therefore, the advanced functions of smartphones allow personnel to carry out certain practices in the clinical environment that may result in a breach in confidentiality and privacy, provoking a proliferation of health data [22–24]. On some occasions, the personnel are not aware of the distraction that occurs when they use smartphones [26], although they acknowledge having observed how other healthcare personnel missed relevant clinical information because of being distracted by their smartphones [20].

In addition, Cho and Lee [27], determined that those nursing students who have a stronger addiction to their mobile phones tend to have a higher possibility of getting distracted during their clinical practicum, noting decreased learning ability [28], and academic performance [19,27]. More than half of nursing students reported that they get distracted during their clinical practicum due to smartphone use [3].

The increasing concern about the adverse effects of overuse of smartphones during clinical practicum implies the need for policies restricting smartphone use while attending to patients. It is important to educate health personnel about the potential risks that can arise from the associated distraction [29]. Therefore, the aim of this study was to analyze the relationship between the level of nomophobia and the distraction associated with the use of smartphones among nursing students during their clinical practicum.

## Methods

### Design

A cross-sectional study was carried out in order to analyze the relationship between the degree of nomophobia and the distraction associated with the use of smartphones by nursing students during their clinical practicum.

### Sample

**Fig 1:**
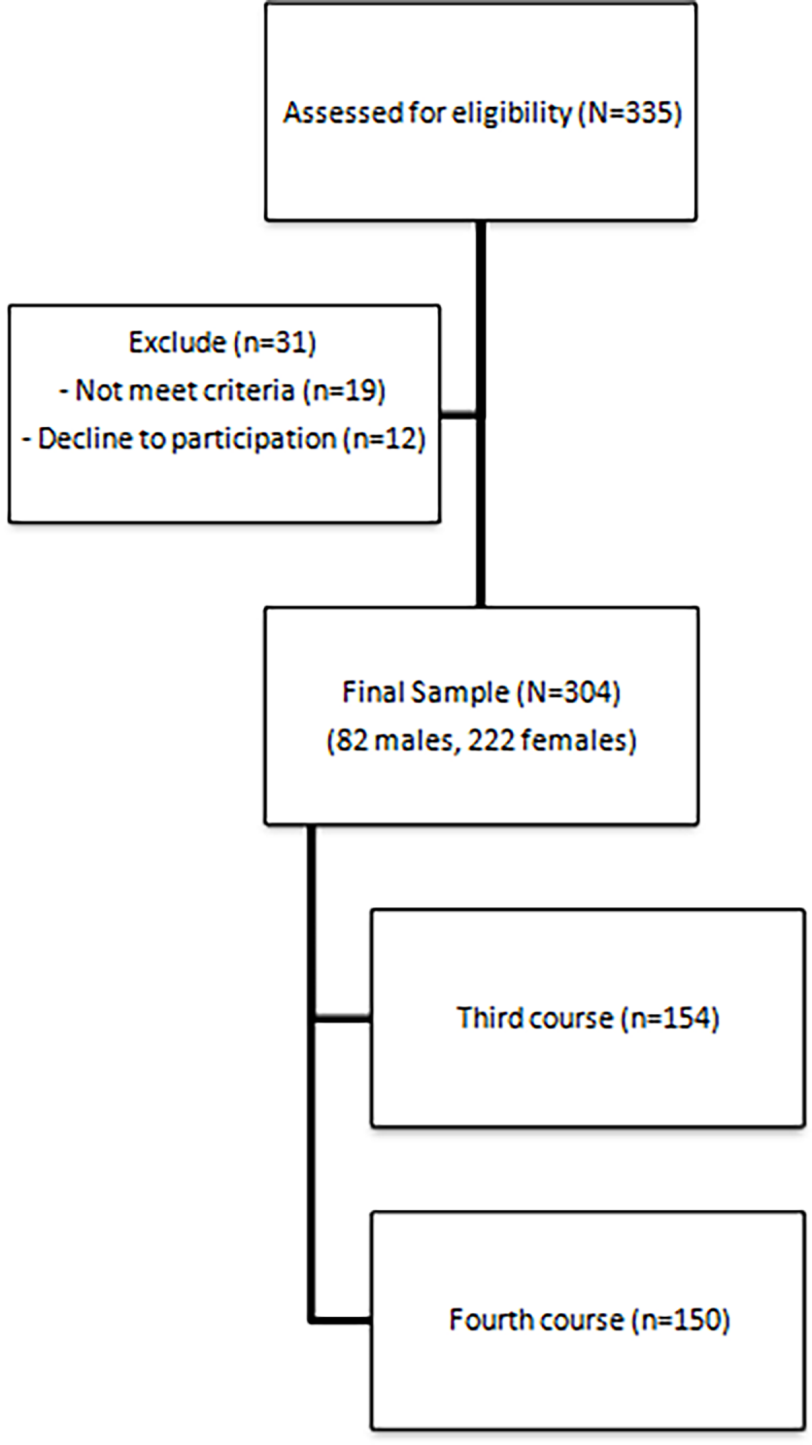
Flow chart of participants.

The general population of study was nursing students, and as a more specific sample, nursing students from the University of Almería were selected. Participants were selected using a convenience sample. The sample was made up of 304 university students studying a nursing degree. The age range of the participants was between 19 and 41 years old. The following factors were considered as inclusion criteria: being enrolled in a nursing degree at the University of Almeria and consenting to take part in the study. Exclusion criteria included not having completed at least six weeks of clinical practicum, or being an exchange student or not have mobile phone (Fig 1).

### Data sources

The nomophobia questionnaire (NMP-Q) was developed and validated by Yildirim and Correia [11]. The questionnaire includes 20 questions using a Likert scale from 1 to 7, with 1 being “totally disagree” and 7 being “totally agree.” These questions are divided into four main themes: not being able to access information (items 1–4); giving up convenience (items 5–9); not being able to communicate (items 10–15) and losing connectedness (items 16–20). The total score is found by adding up the number in each item, which allows for a range of scores from 20 to 140 points. Higher scores correspond to a higher degree of nomophobia. Chronbach’s alpha reliability test was performed, which measures the internal consistency of the scale, and was found to be 0.945. The NMP-Q questionnaire was adapted and validated into the Spanish social and linguistic context, with a Chronbach’s alpha result of 0.927, by Gutiérrez-Puertas, Márquez-Hernández, and Aguilera-Manrique and was used in the sample of Spanish university students [30].

In order to describe these students’ smartphone use, the distraction associated with it, and opinions about phone restriction policies in hospitals, the questionnaire developed and validated by Cho and Lee [3], was used. This questionnaire was developed based on theories of addiction to problematic behaviors, bibliographic reviews on the distraction caused by smartphones and usage policies for smartphones in healthcare environments. The questionnaire consists of 11 items divided into three themes: smartphone use and distraction (1–5), witnessing nurses using smartphones (6–7) and distraction and perception of smartphone restriction policies (8–11). Items 1–7 use a Likert-type scale with options from 1 to 5, 1 = never, 2 = rarely, 3 = sometimes, 4 = usually and 5 = always. Items 8–11 also have a Likert-type scale from 1 to 5, with 1 being “totally disagree” and 5 “totally agree.” The total score is obtained by adding up the number of each of the items. An exploratory factorial analysis was performed with factorization of the main axis with varimax rotation. The results showed an acceptable value of 0.726 on the Kaiser-Meyer-Olkin test. The p value resulting from Bartlet’s sphericity test was lower than 0.001, which indicated that the analysis factor was appropriate. Subsequently, a confirmatory factorial analysis was carried out. The goodness-of-fit index (GFI) of the model was 0.814 and adjusted GFI (AGFI) was 0.701. Factor loadings ranged from 0.398 to 0.833. The correlation coefficients across the latent variables ranged from 0.31 to 0.56, and the model was acceptable.

### Procedures for data collection

Once the study was approved, students in the nursing degree program at the University of Almeria were invited to a meeting. In this meeting, the head researcher explained the objective of this study, as well as the possibility of opting out of the study at any time. Those students who were interested in participating were given an informed consent form, and were reminded of the voluntary nature of their participation, as well as the confidential and anonymous character of their information.

The questionnaires were filled out in one of the university classrooms, during a period of 30–40 minutes. When the session finished, the researcher thanked the participants for their cooperation. Data collection took place between February and May 2017.

### Ethics statement

The study was approved by the Research Committee of the Department of Nursing, Physical Therapy and Medicine at the University of Almeria (nº 8/2016).The committee is composed of expert professors belonging to different areas (nursing, medicine, physiotherapy). The objective of this committee is to supervise, evaluate and approve the research protocols within the department. This Research Committee reports and controls compliance with deontological norms for scientific research, in accordance with the Declaration of Helsinki, the legislation of the European Community, the Spanish State and the Decrees of control and monitoring competence of the Autonomous Community. This committee involve the consideration, discussion and follow-up of the projects by an independent commission of the researcher and the possible sponsor that guarantee the knowledge of the possible predictable risks comparing them with the desirable ones benefits, always prevailing the interest of the subject over the interests of science or society. Also, this committee assesses the considerations taken to protect the privacy of research subjects and the confidentiality of their personal information. In the face-to-face meeting, the main researcher verbally explained the objective of the study, its voluntary nature, and the anonymous and confidential nature of the participants’ data, as well as the possibility of leaving the study at any time. All of the participants were over 18. Before beginning the research, those students who were interested in participating filled out a written informed consent form, as that was one of the criteria to be able to participate in the study. After explaining the study’s purpose and content, written informed consent was provided for all participants.

### Analysis of data

For data analysis, the Statistical Package for the Social Sciences version 24 was used. First, a descriptive analysis of social-demographic variables was carried out. For the qualitative variables, frequencies and percentages were calculated, while, for the quantitative variables, measures of central tendency and measures of dispersion were used. As to the contrast of hypotheses between quantitative and qualitative variables, it was previously verified, using the Kolmogorov-Smirnov test, that the quantitative variables did not follow a normal distribution; thus, non-parametric tests were used. The non-parametric Mann-Whitney U test and Kruskal-Wallis test were used. To compare quantitative variables, Spearman’s correlation test was used. A value of p<0.05 was considered significant.

## Results

### Sample demographic characteristics

Out of the 304 participants, 82 (27%) were male and 222 female (73%). The average age of the participants was 22.77 (SD = 3.65), with a range of ages from 19 to 41 years old. The majority (50.7%) of the students were in their third year of their nursing degree and 49.3% in their fourth year. The social-demographic characteristics of the participants can be seen in Table 1.

**Table 1:** Sample demographic characteristics.

Variable	n	%
Gender:		
Male	82	27
Female	222	73
Academic course:		
Third	154	50.7
Fourth	150	49.3
Daily time spent using a smartphone:		
<1h	48	15.8
1-3h	99	32.6
3-5h	86	28.3
>5h	71	23.4
Most common Smartphone activity:		
Social networking	185	60.9
WhatsApp	279	91.8
Internet searches	131	43.1
Texting	14	4.6
Phone calls	85	28
Listening to music	80	26.3
Watching movies	57	18.8
Playing games	20	6.6
Others	18	5.9
	**M**	**SD**
Age (Range from 19 to 41 years old)	22.77	3.65
Age of beginning smartphone use	13.67	2.21
Frecuency a smartphone use during their clinical practicum	8.36	15.84

### Distraction by smartphone and perception of smartphone restriction policies

Firstly, in regards to smartphone use and distraction, 29.6% (n = 90) of the participants stated that they used a smartphone during their clinical practicum; 45.4% (n = 138) declared that they agreed to have seen another student using their smartphone during their practicum; and 27.3% (n = 83) stated that they had gotten distracted sometimes by watching another student using their smartphone during their practicum.

On the other hand, 23.4% (n = 71) claimed that they had gotten distracted at times by using their smartphone during their clinical practicum. Lastly, 33.9% (n = 103) said that at times they had observed another student being distracted by their smartphone.

Secondly, considering witnessing nurses´ smartphone use and distraction, 37.8% (n = 115) of the participants expressed always seeing nurses use their smartphones at work. 24.7% (n = 75) of the participants admitted sometimes seeing a nurse being distracted by their smartphone during the workday.

Lastly, considering opinions about smartphone restriction policies, 20.1% (n = 61) agreed that they felt uncomfortable when other students used their smartphones during their clinical practicum and 28.6% (n = 87) when the nurses used them. The 36.2% (n = 110) agreed that policies for the restriction of smartphone use by nursing students during their clinical practicum were necessary. Similarly, 39.5% (n = 210) thought that policies to restrict smartphone use by nurses throughout their workday were also necessary. The scores for each item on the questionnaire can be observed in Table 2. No statistically significant differences were found when comparing the dimensions by sex or by age.

**Table 2:** Smartphone use and distraction. Opinion of smartphone restriction policies.

Items	n	%	Range
**Smartphone use and distraction**			
1. Have you used a smartphone during clinical practicum?	3.21*	1.33**	1–5
Never	41	13.5	
Rarely	52	17.1	
Sometimes	84	27.6	
Usually	56	18.4	
Always	71	23.4	
2. Have you witnessed another student using a smartphone during clinical practicum?	4.10*	1.05**	1–5
Never	12	3.9	
Rarely	13	4.3	
Sometimes	25	14.8	
Usually	96	31.6	
Always	138	45.4	
3. Have you been distracted by another student´s use of a smartphone during clinical practicum?	2.85*	1.33**	1–5
Never	66	21.7	
Rarely	56	18.4	
Sometimes	83	27.3	
Usually	57	18.8	
Always	42	13.8	
4. Have you been distracted by own use of a smartphone during clinical practicum?	3.21*	1.33**	1–5
Never	24	13.8	
Rarely	45	14.8	
Sometimes	90	29.6	
Usually	61	20.1	
Always	66	21.7	
5. Have you witnessed another student being distracted by smartphone use during clinical practicum?	3.11*	1.18**	1–5
Never	29	9.5	
Rarely	64	21.1	
Sometimes	103	33.9	
Usually	61	20.1	
Always	47	15.5	
Total Score	16.47*^a^	3.46**	5–25
**Witnessing nurses´smartphone using and distraction**			
6. Have you witnessed another nurse using a smartphone during work?	3.89*	1.14**	1–5
Never	14	4.6	
Rarely	25	8.2	
Sometimes	57	18.8	
Usually	93	30.6	
Always	115	37.8	
7. Have you witnessed nurses being distracted by smartphone use during work?	2.96*	1.31**	1–5
Never	50	164	
Rarely	70	23.0	
Sometimes	75	24.7	
Usually	60	19.7	
Always	49	16.1	
*Total Score*	6.84*^b^	2.15**	5–10
**Opinion about smartphone restriction policies**			
8. I do not want other students to use smartphones during clinical practicum.	2.46*	1.28**	1–5
Totally disagree	96	31.6	
Disagree	60	19.7	
Neither agree nor disagree	87	28.6	
Agree	34	11.2	
Totally agree	27	8.9	
9. I do not want nurses to use smartphones while working.	2.68*	1.30**	1–5
Totally disagree	74	24.3	
Disagree	68	22.4	
Neither agree nor disagree	75	24.7	
Agree	56	18.4	
Totally agree	31	10.2	
10. A policy to restrict nursing students ´smartphone use during clinical practicum is needed.	2.98*	1.31**	1–5
Totally disagree	54	17.8	
Disagree	55	18.1	
Neither agree nor disagree	85	28.0	
Agree	62	10.4	
Totally agree	48	15.8	
11. A policy to restrict nurses ´smartphone use during work is needed.	3.09*	1.32**	1–5
Totally disagree	51	16.8	
Disagree	47	15.5	
Neither agree nor disagree	86	28.3	
Agree	65	21.4	
Totally agree	55	18.1	
Total Score	11.20*^c^	4.23**	4–20

* Mean

**Standard Deviation

^a^ Higher scores correspond to a higher distraction

^b^ Higher scores correspond to a higher distraction

^c^ Higher scores correspond to a higher agreement

### Nomophobia

Considering the results of nomophobia, the average score of each theme can be observed in Table 3. The total average score was 82.39 (SD = 18.63), with statistically significant differences found according to sex (U = 6940.0, z = -3.179, p = 0.001). Females had an average score of 83.62 (SD = 17.50), while males had an average score of 79.04 (SD = 21.16). No statistically significant differences were found when considering age nor age of first smartphone use.

**Table 3:** Average score of each theme of nomophobia.

Items	M	SD	Range
Not being able to access information	17.11	4.23	4–28
Giving up convenience	20.79	5.26	5–35
Not being able to communicate	26.59	6.05	6–42
Losing connectedness	17.87	6.19	5–35
Total Score	82.39^a^	18.63	20–140

^a^ Higher scores correspond to a higher level of nomophobia

### The relationship between the distractions associated with the use of smartphones and nomophobia

While looking for the relationship between the level of distraction associated with smartphone use and nomophobia, we found a low positive correlation between the use of smartphones and giving up convenience (rs = 0.127; p = 0.027), as well as in the total score of nomophobia (rs = 0.113; p = 0.040). In the same way, there was a low positive correlation between opinion about smartphone restriction polices with each of the dimensions of nomophobia and the total score of the questionnaire. These results can be seen in Table 4.

**Table 4:** The relationship between the distraction associated with the use of smartphones and nomophobia.

		Nomophobia				
Distraction by Smartphone		Not being able to access information	Giving up convenience	Not being able to communicate	Losing connectedness	Total Score
Smartphone use and distraction	rs	0.093	0.127	0.093	0.091	0.113
	p	0.104	0.027	0.105	0.113	0.040
Witnessing nurses´smartphone using and distraction	rs	0.062	0.011	0.020	0.024	0.037
	p	0.278	0.843	0.723	0.671	0.524
Opinion about smartphone restriction polices	rs	0.129	0.114	0.144	0.136	0.151
	p	0.025	0.048	0.012	0.018	0.009

## Discussion

The aim of this study was to analyze the relationship between nomophobia levels and the distraction associated with smartphone use among nursing students during their clinical practicum. Firstly, related to smartphone use, in this study, 23.4% of the participants admitted always using their smartphone during their clinical practicum, although not all of them considered smartphone use a distraction. This may be due to the use of smartphones for subjects related to the clinical practicum. In this regard, nursing students feel more secure when using smartphones during their clinical practicum, as doing so allows them to obtain information and resolve any doubts they may have, considering it a support for making clinical decisions [31]. Likewise, almost half of the participants stated that they had seen other students use their smartphones during their practicum. Along the same lines, Cho and Lee [3], found even higher numbers. In their study, 46.2% of nursing students acknowledged that they used their smartphone sometimes, while 63.2% had observed how other nursing students had used them too. Those nursing students who admitted being distracted by smartphone use were in the minority, just like other studies have shown [3,15,16]. However, they had observed how their classmates get distracted using their smartphones. This data concurs with that reported by other researchers [3,16].

Different studies show to what extent nursing professionals acknowledge using their smartphones while at work [15,17], and also to what extent they see nursing professionals using them in a clinical setting [3]. This data goes along with the data obtained in this study. This elevated use of smartphones may indicate that nursing professionals have a need to share their emotional stress with family and friends, in order to alleviate the emotional fatigue associated with this type of work, characterized by rotating shifts, high patient demand, and low quality of sleep [15,32] feeling a need to let off steam or unwind through smartphone use [33]. Another study determined that this type of use may be due to “fear of missing out” therefore, they need to stay connected to alleviate their social anxiety [34]. Various studies show that nurses do not acknowledge getting distracted by smartphones at work [15,17,35]. This may be due to the fact that the distraction caused by smartphones can be perceived as a productive break, allowing them to satisfy their personal needs, which has a positive effect [35]. It also may be due to the fact that nurses are not aware of their own shortcomings or errors that happen while using a smartphone, or they do not think that smartphones distract them [15,24].

With regards to the need for establishing policies that restrict smartphone use in the clinical setting, in this study, 36.9% of the nursing students agreed that it is necessary to limit smartphone use among nursing professionals and students throughout their workday. In the same vein, the study performed by Cho and Lee [3], found that 29.2% of nursing students agree to establishing smartphone use policies. However, according to Bautista and Lin [17], nursing professionals do not agree that it is necessary to establish smartphone use restriction policies in a clinical setting. They maintain that personal smartphone use can even be of benefit to patient care. These results contrast with the ones found in this study. More specifically, in this study, the correlation between nomophobia and the need to establish restriction policies was very weak. The nursing students who showed a higher level of nomophobia agreed that the development of restriction policies in the clinical setting was necessary. However, other studies indicate that nursing students who reported higher levels of smartphone addiction did not agree that it was necessary to set up policies that regulated smartphone use in a clinical setting [3,27].

On the other hand, regarding nomophobia, the results indicate a correlation between smartphone use and nomophobia. Along the same lines, Kaur and Sharma [18], found a moderate correlation between smartphone use patterns and the risk of developing nomophobia. As in other studies, the results obtained showed that females exhibited higher levels of nomophobia than males [12,14], which may be related to the type of communication each gender uses. In this case, women tend to get more involved in and maintain more active relationships than men [36]. Either way, further studies about gender would be necessary to prove the individual tendency to develop nomophobia [14].

With regards to the relationship between nomophobia and age, no statistically significant differences were found. In line with our study, several authors claim that they found no differences in regards to age [14,37,38], seeing as nomophobia can be developed at any age [10]. In this study, this may be due to the fact that the range of ages in the study is relatively small. The association between age and nomophobia has not been clarified, and thus, it would be necessary to carry out further studies with participants of a wider range of ages [14].

The results of this study should be regarded while keeping in mind a series of limitations. The sample was selected by convenience, which may affect the generalization of the results. Secondly, the results could be found to be biased because of the socially desirable bias. Some authors, such as Polit and Beck [39], state that some students may obscure their responses by giving answers that reflect their social values or professional expectations. Another possible bias of the study was in the way the students measured their smartphone use by simply answering a question on the questionnaire and not using objective measurement methods (for example, through applications on their smartphones) [40]. The occupation of the participants’ parents has not been taken into consideration, and it may be relevant to determine if their social environment could have an impact on their smartphone use, as previous studies indicate that people with a lower education level have a higher risk of smartphone addiction [41].

Lastly, the lack of research available on this topic makes a proper discussion of the topic difficult, but at the same time, highlights the novel and interesting nature of this research, having rarely been studied in depth before.

## Conclusions

Nursing students who show high levels of nomophobia also regularly use their smartphones throughout their clinical practicum. However, these students are aware of the need to create policies that regulate smartphone use in the clinical context. It is necessary to address the issue of dependence on smartphones and the consequences that the distraction they create can have in a clinical setting. In the same way, it is necessary to introduce smartphone use regulations and determine their impact on nursing professionals and students in an educational setting, as well as in a healthcare setting.

## Supporting information

S1 Data Base Database on distraction by mobile phone and nomophobia. (SAV)
